# Supplementary figures and images for: Nuclease escape elements protect messenger RNA against cleavage by multiple viral endonucleases
Source: PLoS Pathog. 2017 Aug 25;13(8):e1006593. doi: 10.1371/journal.ppat.1006593 (PMC5589255; doi:10.1371/journal.ppat.1006593)

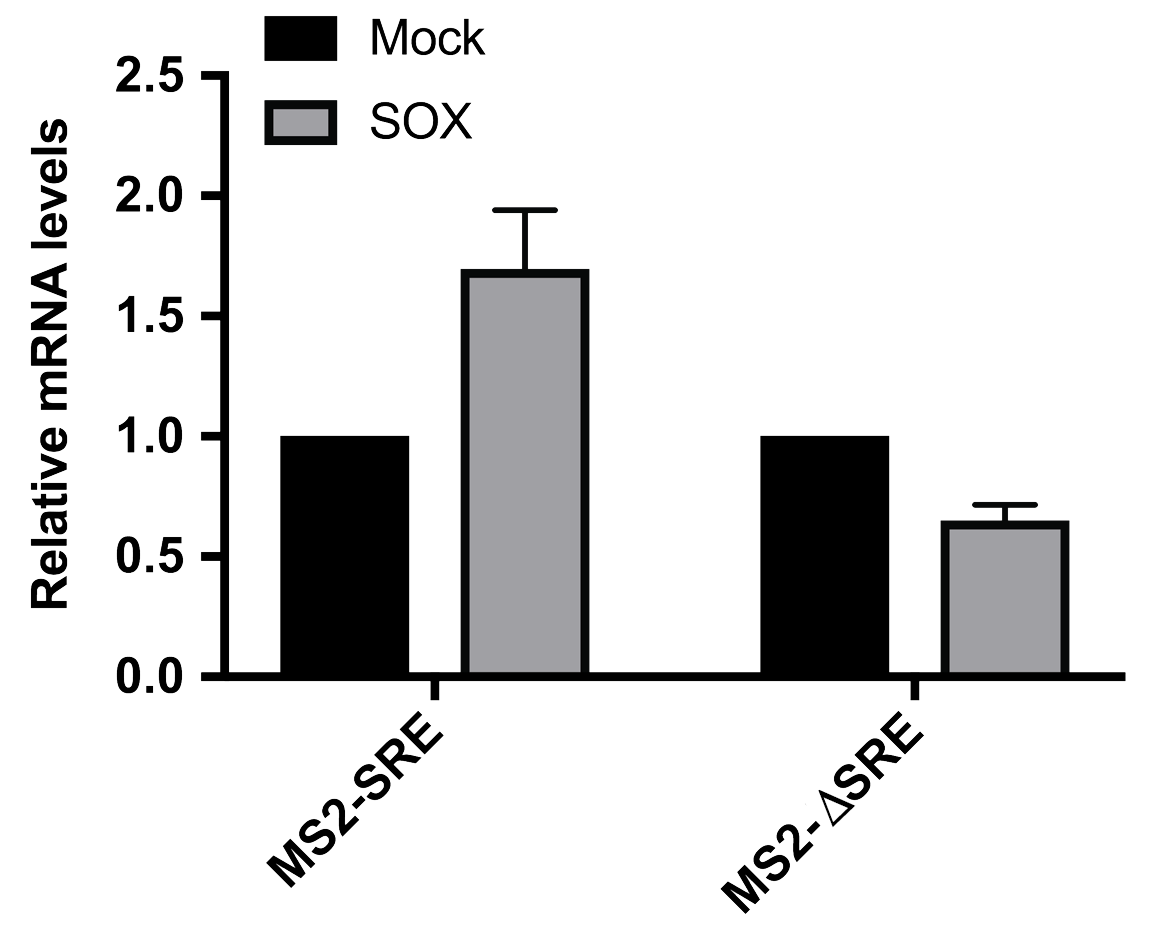

Supplement: S1 Fig — After 24 h, total RNA was harvested and subjected RT-qPCR to measure reporter mRNA levels. (TIF) [file ppat.1006593.s001.tif]

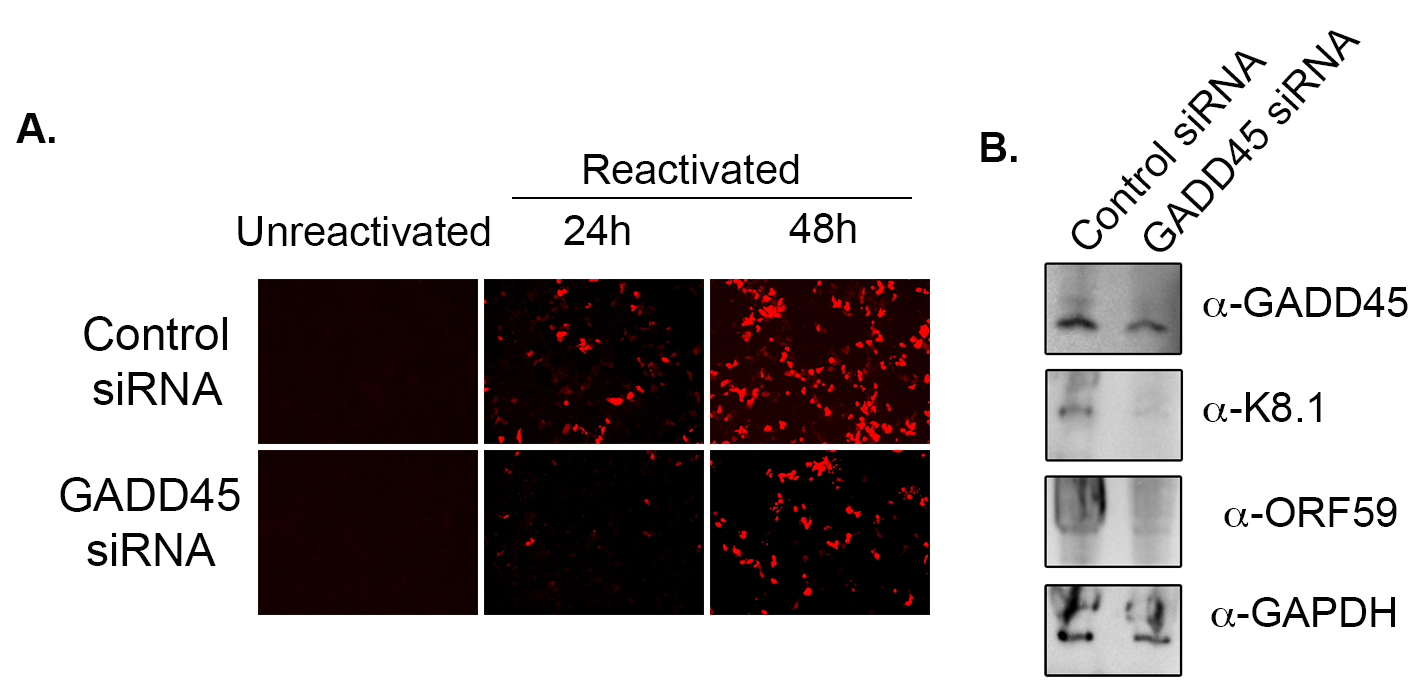

Supplement: S2 Fig — (A) iSLK.219 cells were treated with siRNAs targeting GADD45B (or control non-target siRNAs) for 48h. Cells were then reactivated with doxycycline and sodium butyrate for 24 or 48h, fixed, and reactivation efficiency was monitored by expression of red fluorescent protein, which is expressed from the viral genome under the control of the lytic PAN promoter. (B) After siRNA treatment and reactivation as described in A, cell lysates were subjected to western blotting to measure protein levels of GADD45B, the KSHV proteins K8.1 and ORF59, and GAPDH (as a loading control). (TIF) [file ppat.1006593.s002.tif]

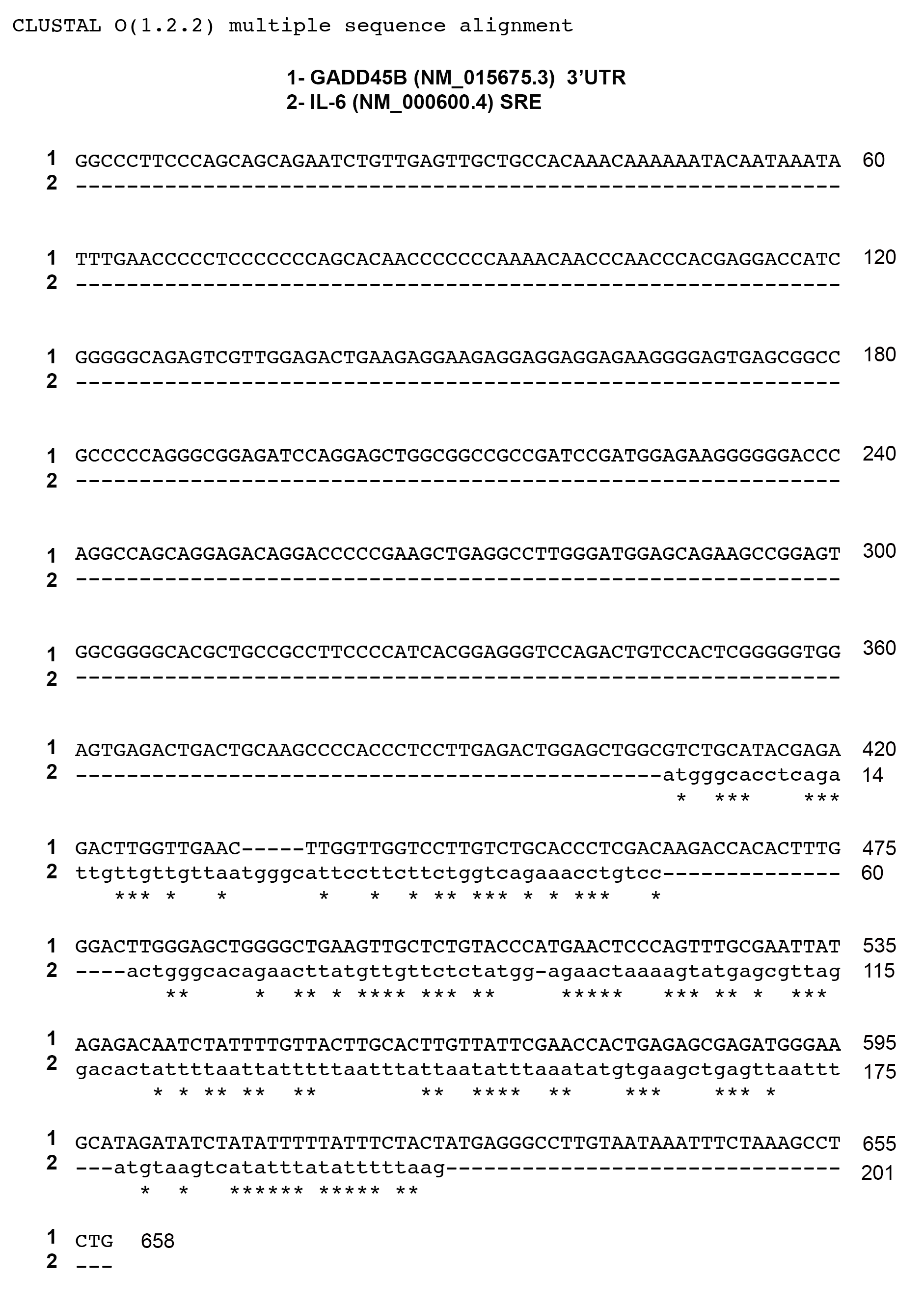

Supplement: S3 Fig — (TIF) [file ppat.1006593.s003.tif]

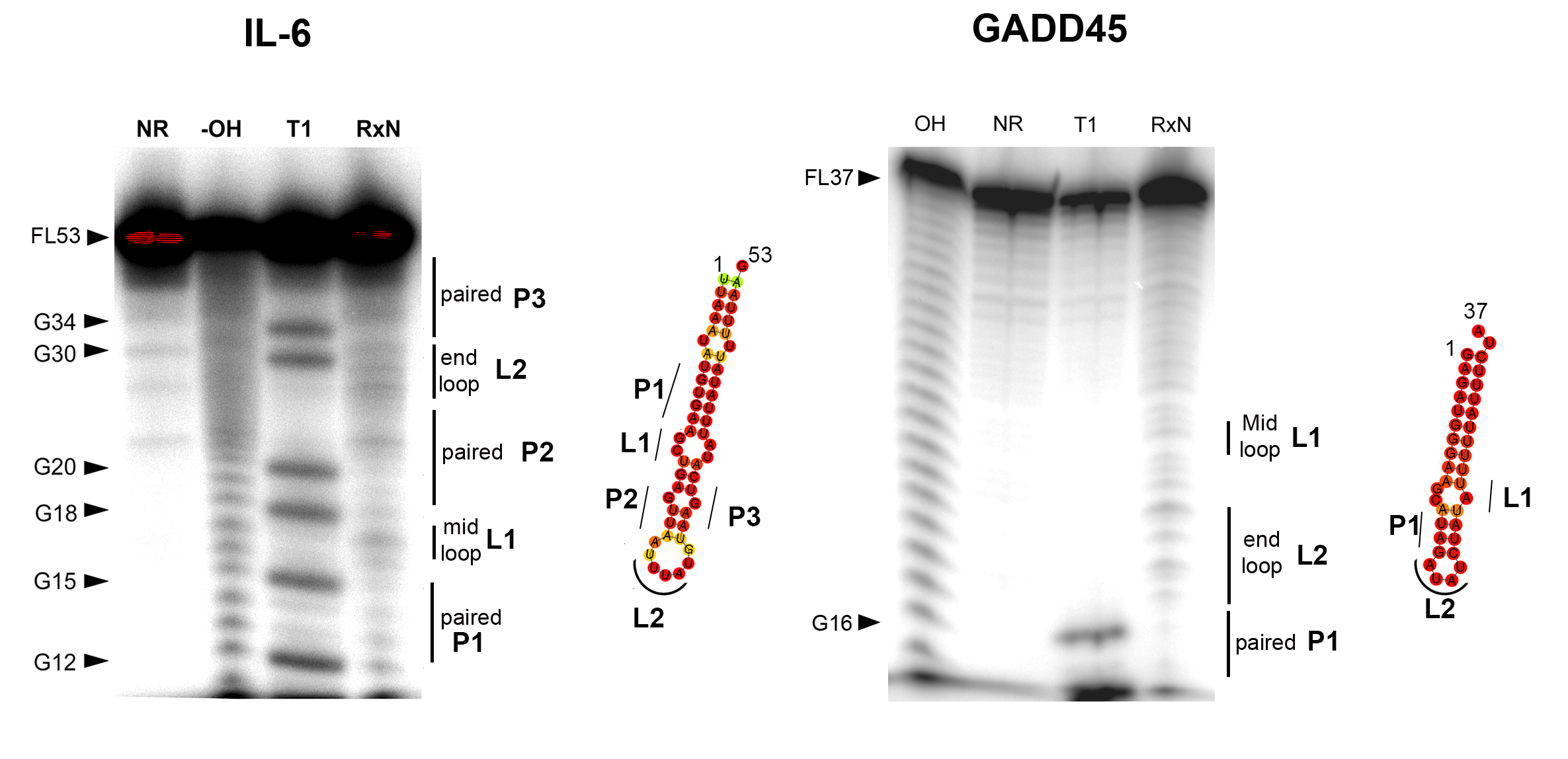

Supplement: S4 Fig — 32P-labeled RNA (NR, no reaction) and products resulting from partial digestion with nuclease T1 (T1; cuts after G residues), partial digestion with alkali (-OH), and spontaneous cleavage during a 24h incubation are shown. Product bands corresponding to G residues (generated by T1 digestion) are labeled with black arrows. Predicted paired or unpaired residues are marked on the right of each gel and are shown on the RNA fold diagrams. (TIF) [file ppat.1006593.s004.tif]

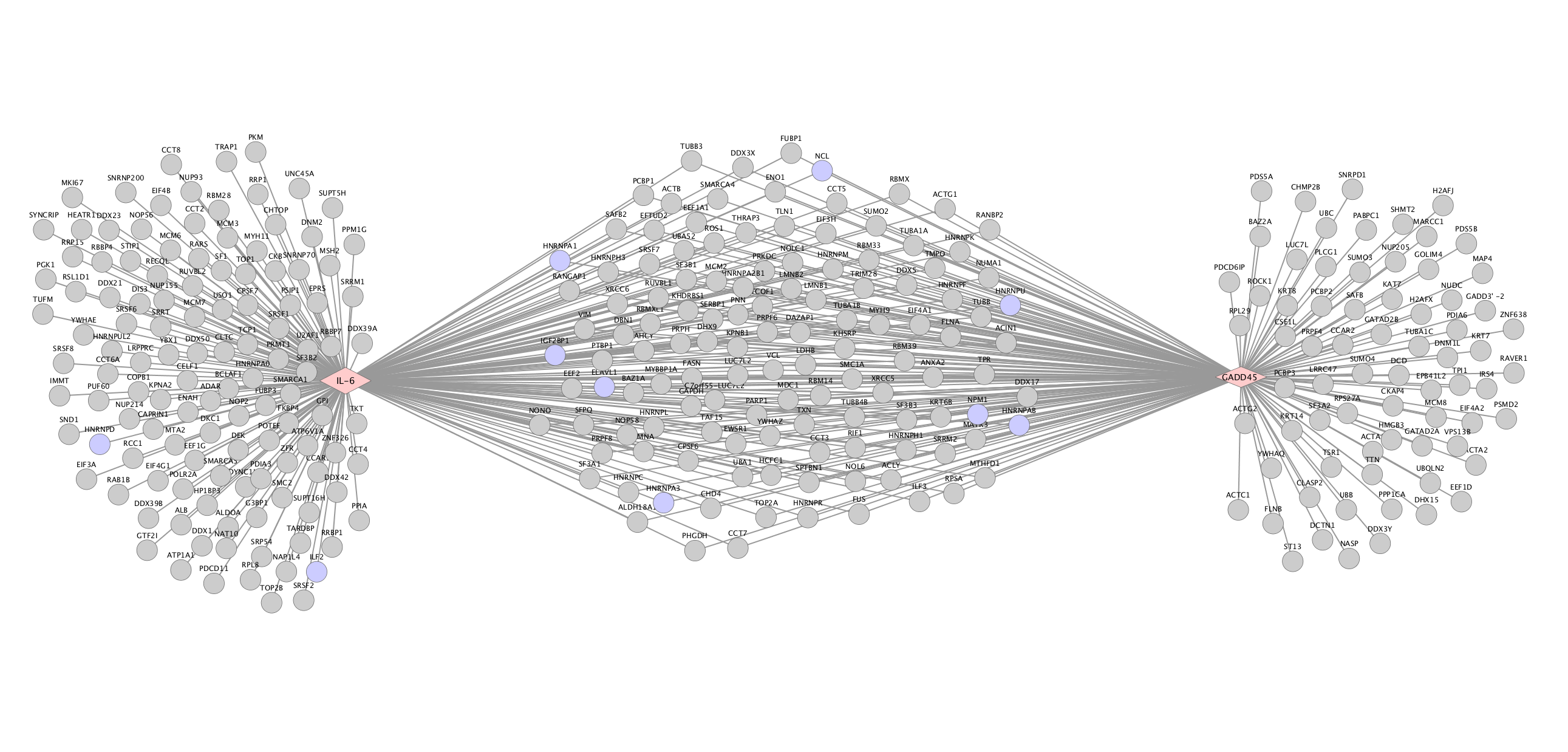

Supplement: S5 Fig — Purple nodes represent proteins that were previously identified using an in vitro pulldown/MS-based assay [27]. (PNG) [file ppat.1006593.s005.png]
